# Correlates of evolutionary rates in the murine sperm proteome

**DOI:** 10.1186/s12862-018-1157-6

**Published:** 2018-03-27

**Authors:** Julia Schumacher, Holger Herlyn

**Affiliations:** Institute of Organismic and Molecular Evolution, Anthropology, Johannes Gutenberg University, Mainz, Germany

**Keywords:** Protein evolution, Tissue specificity, Protein essentiality, Sperm proteins, Protein-protein interactions, Gene age, Multifunctionality, Paralogs, Gene compactness, dN/dS

## Abstract

**Background:**

Protein-coding genes expressed in sperm evolve at different rates. To gain deeper insight into the factors underlying this heterogeneity we examined the relative importance of a diverse set of previously described rate correlates in determining the evolution of murine sperm proteins.

**Results:**

Using partial rank correlations we detected several major rate indicators: Phyletic gene age, numbers of protein-protein interactions, and survival essentiality emerged as particularly important rate correlates in murine sperm proteins. Tissue specificity, numbers of paralogs, and untranslated region lengths also correlate significantly with sperm genes’ evolutionary rates, albeit to a lesser extent. Multifunctionality, coding sequence or average intron lengths, and mean expression level have insignificant or virtually no independent effects on evolutionary rates in murine sperm genes. Gene ontology enrichment analyses of three equally sized murine sperm protein groups classified based on their evolutionary rates indicate strongest sperm-specific functional specialization in the most quickly evolving gene class.

**Conclusions:**

We propose a model according to which slowly evolving murine sperm proteins tend to be constrained by factors such as survival essentiality, network connectivity, and/or broad expression. In contrast, evolutionary change may arise especially in less constrained sperm proteins, which might, moreover, be prone to specialize to reproduction-related functions. Our results should be taken into account in future studies on rate variations of reproductive genes.

**Electronic supplementary material:**

The online version of this article (10.1186/s12862-018-1157-6) contains supplementary material, which is available to authorized users.

## Background

The spermatozoon is a highly specialized cell type indispensable for male fitness. Different types of postmating sexual selection are thought to drive the diversification of sperm anatomy and function across taxa. For instance, sperm competition, the competition between sperm from several males for fertilization of a female’s ova [1], is considered to increase sperm viability in *Drosophila* [2] and numbers of sperm produced in mice [3, 4]. In primates, sperm midpiece volume varies depending on mating systems and thus presumably in response to different levels of sexual selection [5] (for rodents, see [6]). Furthermore, sperm competition and other types of sexual selection, such as cryptic female choice (see, e.g., [7]) and sexual conflict (reviewed in [8]), may exert selective pressures not only on sperm form or production, but also on the proteins constituting these cells. Accordingly, adaptive evolution of male reproductive proteins has been observed in a wide range of taxa (see, e.g., [9–12]). However, studies with proteome-wide perspectives demonstrated that despite the rapid evolution of certain reproduction-related genes, the majority of male reproductive proteins are conserved [13, 14]. This variation in sequence evolution of male reproductive proteins is moreover characterised by temporal and spatial compartmentalization of sperm or testis proteomes with higher rates in later steps of spermatogenesis and in proteins acting proximate to fertilization (see, e.g., [15–18]). Dorus et al. [13] hypothesized that the strong conservation of many sperm proteins might rely on either their involvement in critical cellular functions or their expression in nonreproductive tissues, which generates pleiotropic constraints. Corresponding to the second prediction of Dorus et al. [13], other authors indeed found rate acceleration of testis-specific genes in *Drosophila* compared with genes also expressed in other tissues [19]; in the mouse sperm proteome, however, testis overexpression was insufficient to explain evolutionary rate variation [16].

Hence, although it may be assumed that all protein-coding genes expressed in sperm cells could potentially experience sexual selection, adaptive evolution affects only a relatively small fraction of them. The above mentioned studies were able to partially explain rate variations of male reproductive proteins. However, the influence of a larger set of potential rate determinants on the evolution of mammalian sperm proteins has not yet been studied.

In evolutionary biology, the quest for such rate determinants has long been a central issue and several correlates of evolutionary rates have been identified (reviewed in, e.g., [20, 21]): The evolutionary rate of a protein is thought to be mainly shaped by its level of essentiality and functional constraint [22]. Essentiality manifests in the fitness effect of gene deletion and has been shown to correlate negatively with evolutionary rates (see, e.g., [23]). Functional constraint refers to the compatibility of substitutions with a protein’s function (see, e.g., [22]), which, however, is more difficult to quantify than essentiality. Still, several correlates of evolutionary rates, such as connectivity in protein-protein interaction (PPI) networks [24], multifunctionality [25], and expression breadth [26, 27] have been established, all of which might relate to pleiotropic [28, 29] and/or structural and functional constraints [30, 31]. Moreover, other variables including phyletic gene age [32, 33], gene compactness [34], expression level [35–37] or numbers of paralogs [38, 39] have been identified as substantial rate indicators of protein-coding genes.

In the present study we aim to uncover factors influencing rate variations of sperm proteins. We intend to unravel the relative importance of several proposed variables as correlates of sperm proteins’ evolutionary rates. Based on a published murine sperm proteome [40] we used zero-order and partial rank correlations to disentangle the effects of individual gene properties on dN/dS. This latter measure is the ratio of nonsynonymous (dN) and synonymous substitution rates (dS), with dN/dS = 1, < 1, and > 1 indicating neutral evolution, purifying, and positive selection, respectively. We included essentiality, multifunctionality, number of PPIs, tissue specificity (τ), mean expression level inferred over 22 mouse tissues, phyletic gene age, number of paralogs, and different measures of gene compactness (coding sequence (CDS) length, 5′ and 3′ untranslated region (UTR) length, average intron length) as potential correlates of sperm proteins’ dN/dS values. Additionally, we retrieved gene ontology (GO) information for sperm protein groups with different rates of sequence evolution. Based on our results, we propose a model for sperm protein evolution. Our findings underscore the relevance of tissue- or cell type-specific analyses of putative rate determinants.

## Methods

Ensembl Gene IDs corresponding to proteins expressed in murine epididymal sperm according to Chauvin et al. [40] were identified using Uniprot’s ID mapping tool (19th march 2015) and Ensembl Biomart version 79 (for details, see Additional file 1: Supplementary Methods).

For each sperm gene, we extracted pairwise dN/dS estimates from the orthologues view pages in Ensembl version 79. On these pages, dN/dS values potentially biased by dS saturation are hidden (http://mar2015.archive.ensembl.org/info/genome/compara/homology_method.html), so that our data should be largely unaffected by such bias. We considered dN/dS values which had been estimated between 1-to-1 orthologues of mouse (*Mus musculus*; genome assembly GRCm38.p3) and rat (*Rattus norvegicus*; genome assembly Rnor_5.0) using CodeML from the PAML package [41].

Descriptions of how we determined the number of paralogs, CDS, average intron as well as 5′ and 3′ UTR length for each gene can be found in Additional file 1: Supplementary Methods.

Genes coding for the sampled sperm proteins were classified into six age groups corresponding to their earliest phyletic origin and coded as numbers descending towards younger ages: cellular organisms (6), Eukaryota (5), Fungi/Metazoa (4), Metazoa (3), Chordata (2), Mammalia (1). Age classifications were taken from supplementary data of Chen et al. [42] and had been estimated applying a bit score cutoff of 80 in FASTA (for more details, see supplementary methods of [42]).

Numbers of direct and indirect PPI partners relating to Swiss-Prot accession numbers were extracted from downloadable data of I2D (Interologous Interaction Database; http://ophid.utoronto.ca/; [43]) version 2.9 (for details, see Additional file 1: Supplementary Methods).

For each gene, the tissue specificity index τ [44] and the mean expression level were gathered from supplementary data of Kryuchkova-Mostacci and Robinson-Rechavi [39]. The index τ ranges from 0 to 1, with larger values indicating higher tissue specificity [44]. Both τ and mean expression level had originally been calculated based on ENCODE [45] expression data comprising 22 mouse tissues [39].

In order to measure multifunctionality, we assigned numbers of biological processes per protein [46] from GOSlim generic annotations to each Swiss-Prot ID (for details, see Additional file 1: Supplementary Methods).

MGI (Mouse Genome Informatics) IDs corresponding to Ensembl Gene IDs were identified via Ensembl Biomart version 79. Based on downloadable data from MGI (version 5.22; http://www.informatics.jax.org/; [47]; state: 19th August 2015), we determined the knockout phenotype for each gene in our dataset. We exclusively considered homo- or – in case of X-chromosomally encoded genes – hemizygous null alleles (with allele attributes containing the term “Null/knockout”) generated by homologous recombination (allele type “Targeted”). Furthermore, we incorporated only alleles affecting single genes. Essential genes were those associated with any of the MP (Mammalian Phenotype) IDs summarized under “preweaning lethality” (MP:0010770) or “lethality at weaning” (MP:0008569). All other genes with known phenotypes conforming to the criteria outlined above were classified as “nonessential”. Genes without such known phenotypes were left unregarded in analyses involving essentiality.

Our statistical analyses conform to the principles applied in related exploratory studies (see, e.g., [34, 48, 49]). Spearman’s rank correlations and partial Spearman’s rank correlation were conducted with MATLAB version R2015b. Genes with lacking information regarding any variable were removed from the dataset so that we included only those for which all variables were available. As many genes had to be excluded from analyses due to missing information concerning knockout phenotypes, we computed all correlations twice (except for those comprising essentiality), in a dataset with (681 proteins) and in a dataset without the essentiality variable (1557 proteins).

To assess potential functional trends in dependence of evolutionary rates, we divided our protein sample (without essentiality data; n = 1557) into three equally sized bins of 519 proteins according to their genes’ dN/dS estimates: bin 1 (“low dN/dS”; dN/dS ≤ 0.0362); bin 2 (“medium dN/dS”; dN/dS 0.03633–0.12561); bin 3 (“high dN/dS”; dN/dS ≥ 0.12574). For each bin, we performed an enrichment analysis by functional annotation clustering based on GOTERM_BP_FAT using the Database for Annotation, Visualization and Integrated Discovery (DAVID; version 6.7; https://david.ncifcrf.gov/home.jsp; [50, 51]). To this end, we entered Ensembl Gene IDs, all of which could be matched to DAVID IDs, and specified the classification stringency as “high”.

## Results and discussion

We compiled a set of proteins expressed in mouse epididymal sperm [40] and collected the pairwise dN/dS estimate with rat for each coding gene as well as several variables which had previously been shown to correlate with evolutionary rates. The potential correlates of dN/dS comprised gene essentiality, multifunctionality, number of PPIs, expressional tissue bias (τ), mean expression level, phyletic gene age, number of paralogs, and several measures of gene compactness (CDS length, 5′ and 3′ UTR length, average intron length). We included only genes for which all variables were available, restricting our rank correlation analyses to 681 genes. The most limiting factor was the accessibility of knockout data to evaluate a gene’s essentiality for survival: such data existed for less than half of the genes for which all other variables were obtainable. Due to this confined availability of knockout data we recalculated the correlations without essentiality in a set of 1557 genes. Results of Spearman’s rank correlations were largely similar in both datasets, showing significant correlations of most variables with dN/dS (Table 1). Only CDS and intron length did not correlate significantly with dN/dS values and the signs of their correlation coefficients differed between the datasets incorporating 681 or 1557 proteins.

**Table 1:** Spearman’s rank correlations between dN/dS and each gene property

Gene properties	ρ with dN/dS (n = 681)	ρ with dN/dS (n = 1557)
5′UTR	−0.172***	−0.132***
3′UTR	−0.223***	−0.141***
CDS	−0.014 (ns)	0.044 (ns)
intron	−0.017 (ns)	0.023 (ns)
multi	−0.099**	−0.051*
τ	0.293***	0.228***
PPI	−0.305***	−0.293***
essentiality	−0.303***	NA
ex.lev.	−0.259***	−0.246***
para	−0.154***	−0.105***
age	−0.268***	−0.214***

*age* Phyletic age of genes, *multi* Multifunctionality (number of biological processes per protein according to GOSlim generic), *PPI* Number of PPIs, *para* Number of paralogs, *CDS* Coding sequence length; *intron* average intron length, *3′UTR* Length of 3’ UTR, *5′UTR* Length of 5′UTR, *essentiality* essentiality for survival, 0 = nonessential, 1 = essential, τ tissue specificity index, *ex.lev.* mean mRNA expression level, *NA* Not applicable. Significance: *p < 0.05; **p < 0.01; ***p < 0.001; *ns* Nonsignificant

**Fig. 1:**
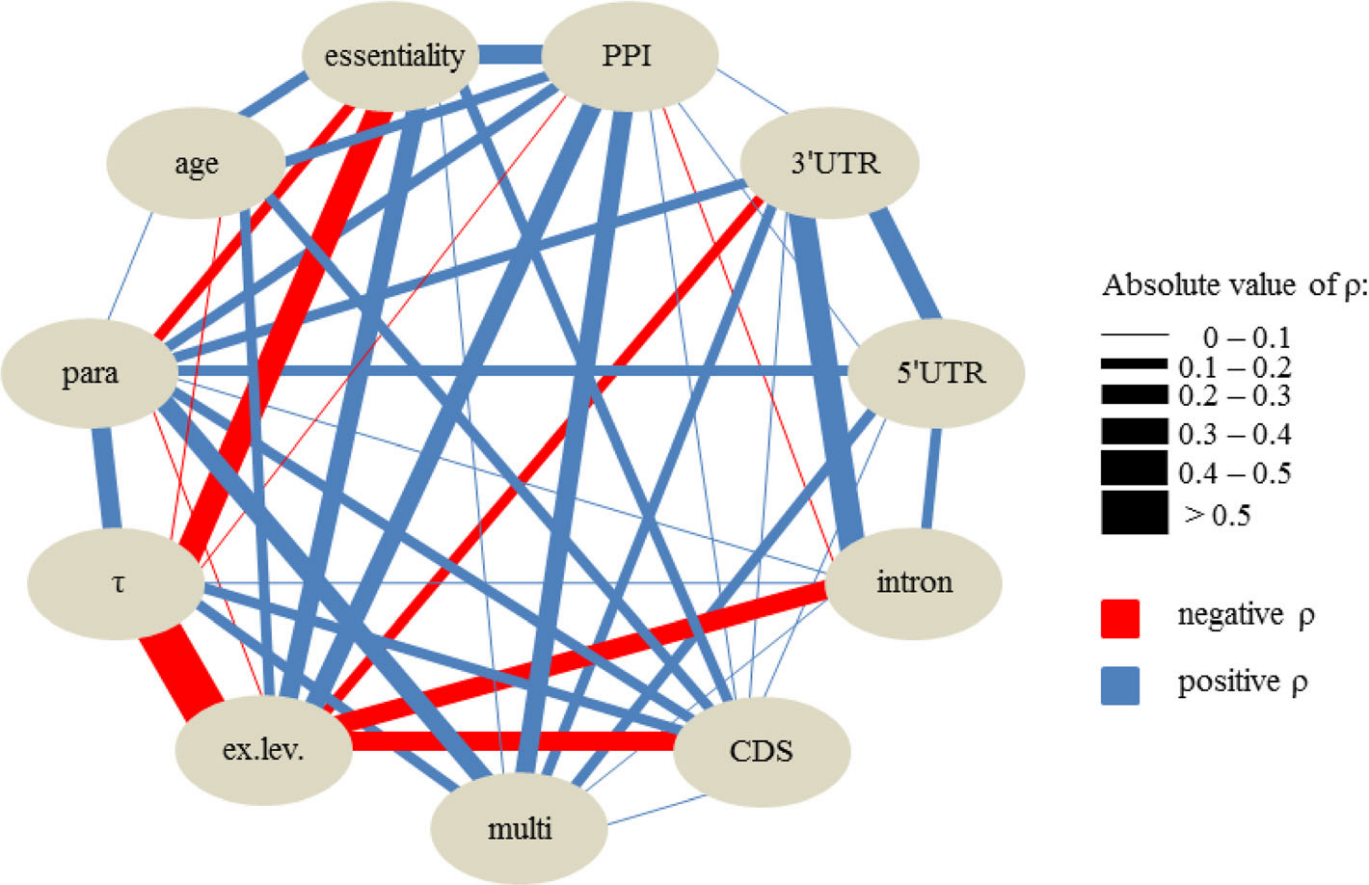
Spearman’s rank correlation between all variable pairs, excluding dN/dS. Edges represent correlation coefficients (ρ) between two properties. All correlations were calculated in the dataset of 1557 proteins, apart from those involving essentiality, which are calculated for 681 proteins. Only correlations significant with *p* < 0.05 are depicted. See legend of Table 1 for descriptions of the biological variables: 3′UTR, 5′UTR, age, essentiality, ex.lev., multi, para, PPI, CDS, intron, and τ

**Fig. 2:**
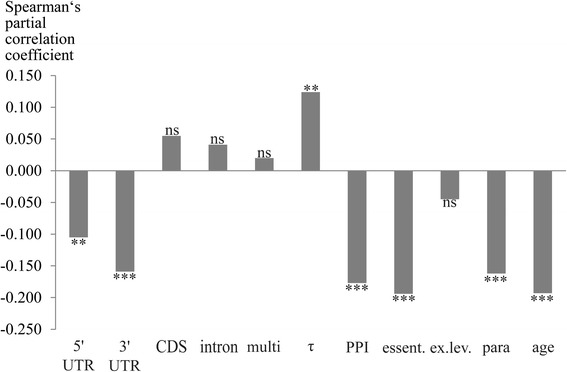
Spearman’s partial rank correlation between dN/dS and each variable, while controlling for all other variables including essentiality (number of genes = 681). essent., essentiality for survival, 0 = nonessential, 1 = essential. See legend of Table 1 for descriptions of the biological variables: age, ex.lev., multi, para, PPI, τ, CDS, intron, 3′UTR, and 5′UTR; significance: **p < 0.01; ***p < 0.001; ns, nonsignificant

**Fig. 3:**
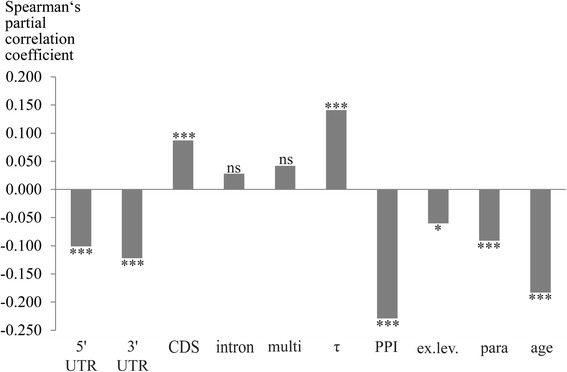
Spearman’s partial rank correlation between dN/dS and each variable, while controlling for all other variables excluding essentiality (number of genes = 1557). See legend of Table 1 for descriptions of the biological variables: age, ex.lev., multi, para, PPI, τ, CDS, intron, 3′UTR, and 5′UTR; significance: *p < 0.05; ***p < 0.001; ns, nonsignificant

Due to interdependencies among the gene properties (Fig. 1; Additional file 1: Figure S1) we employed partial rank correlations to determine the relative strength of correlation for each variable with dN/dS; thereby, we controlled for all remaining gene properties. Partial rank correlations have been used previously to examine rate determinants in various taxa (see, e.g., [34, 49, 52]). Figures 2 and 3 depict partial rank correlation coefficients and *p* values for each studied variable with pairwise dN/dS estimates. Whether essentiality was included or excluded, most results remained qualitatively unchanged (Figs. 2 and 3).

Additionally, we computed partial rank correlations between each gene property and either dN or dS calculated for orthologous sequences of mouse and rat (see Additional file 1: Supplementary Methods). Results of these analyses demonstrate that the patterns observed for dN/dS are either driven by dN or a combination of dN and dS, but none of the significant partial correlations with dN/dS can solely be traced back to the impact of dS (see Additional file 1: Tables S1 and S2). Furthermore, results of partial rank correlations including dN/dS remained qualitatively unchanged when obtained in two datasets (with or without the essentiality variable) after exclusion of genes with signals of positive selection according to CodeML analyses (see Additional file 1: Supplementary Methods and Table S3). It can, thus, overall be concluded that none of our observations is the result of an overabundance of positively selected genes.

### Gene compactness

#### UTR and intron length

Liao et al. [34] reported that of the factors analysed in their study, gene compactness in terms of UTR and average intron length was the most important determinant of evolutionary rate in murine protein-coding genes. They found significant negative correlations between dN/dS and both UTR length and average intron length, whereas the zero-order correlation between evolutionary rate and CDS length was nonsignificant. In our sperm gene dataset we also detected significant negative partial correlations between pairwise dN/dS estimates and both 5′ (681 genes: Spearman’s partial ρ = −0.105, *p* < 0.01; 1557 genes: Spearman’s partial ρ = −0.101, *p* < 0.001) and 3′ UTR lengths (681 genes: Spearman’s partial ρ = −0.159, *p* < 0.001; 1557 genes: Spearman’s partial ρ = −0.122, *p* < 0.001) (Figs. 2 and 3). Untranslated regions are crucial for posttranscriptional regulation implemented via diverse mechanisms most of which utilize *cis*- and *trans*-acting elements (see e.g., [53, 54]). Against this background, genes with tightly adjusted expression patterns may have a tendency to contain more binding sites for regulatory molecules, which might entail longer UTRs. Accordingly, Cheng et al. [55] uncovered that human and mouse genes targeted by more distinct types of miRNAs (microRNAs) have longer 3′ UTRs and evolve at lower rates. This pattern applies to genes with critical functions whose mRNAs’ translation must be precisely adjusted to ensure correct temporal and spatial expression. Housekeeping genes, however, are less regulated by miRNAs and have short 3′ UTRs, but are also slowly evolving [55]. Such opposing regulation patterns via UTR binding elements might have an impact on the correlations between UTR lengths and dN/dS. These correlations could presumably be stronger or weaker in different subsamples of genes with or without spatial or temporal expression variation, according to the patterns outlined above [55].

In contrast, average intron length showed no significant partial correlations with dN/dS in the current study (681 genes: Spearman’s partial ρ = 0.041, *p* > 0.05; 1557 genes: Spearman’s partial ρ = 0.028, *p* > 0.05) (see Figs. 2 and 3). This result disagrees with findings of previous investigations on mouse genes which revealed significant negative correlations [34, 56]. But as both partial and zero-order correlations (see Table 1, Figs. 2 and 3) were nonsignificant, average intron length does not seem to be an important rate indicator in our sperm protein dataset.

#### CDS length

Partial correlation between CDS length and dN/dS was significant in the larger dataset of 1557 genes (Spearman’s partial ρ = 0.087, *p* < 0.001), but nonsignificant in the more restricted set (n = 681; Spearman’s partial ρ = 0.055, *p* > 0.05). In both cases, however, the partial correlation coefficient indicated a positive relationship between evolutionary rate and sequence length (see Figs. 2 and 3). The interplay of these two variables has been studied in different taxa, but the results were contradictory: While some authors detected no significant correlation between rates of sequence evolution and protein or CDS length (see, e.g., [34]), others reported significant positive (see, e.g., [49, 57, 58]) or negative correlations (see, e.g., [39, 59]). Chang and Liao [49] proposed that the positive correlation between dN/dS and CDS length might rely on lower domain density and thus lower percentage of functionally important residues in longer proteins. However, they concluded that this explanation is insufficient to fully account for this correlation, at least in the flagellated algae studied [49]. Based on the current data we are unable to definitely resolve the relation between CDS lengths and evolutionary rates of murine sperm proteins. But regardless whether CDS lengths and evolutionary rates are related, other gene properties studied herein are considerably more strongly correlated with dN/dS (see Figs. 2 and 3).

### Pleiotropy and essentiality

We measured different aspects of pleiotropy: degree of expressional tissue bias (τ), numbers of PPIs, and multifunctionality. The latter, defined as the number of biological processes a protein is involved in [46], correlated negatively with evolutionary rates of human genes in a study by Podder et al. [25]. We found nonsignificant positive partial correlations (681 genes: Spearman’s partial ρ = 0.020, *p* > 0.05; 1557 genes: Spearman’s partial ρ = 0.042, *p* > 0.05) (Figs. 2 and 3) between multifunctionality and dN/dS, while the corresponding zero-order correlations were negative and significant (681 genes: Spearman’s ρ = −0.099, *p* < 0.01; 1557 genes: Spearman’s ρ = −0.051, *p <* 0.05) (Table 1). Together, these results illustrate that multifunctionality and sequence evolution of murine sperm proteins are largely unrelated when controlling for all other gene properties.

In contrast, we observed a positive partial correlation between tissue specificity index τ and dN/dS, which was significant in both datasets (681 genes: Spearman’s partial ρ = 0.124, *p* < 0.01; 1557 genes: Spearman’s partial ρ = 0.141, *p* < 0.001) (Figs. 2 and 3). This index ranges between 0 and 1, with higher values indicating more tissue-biased expression [44]. Our observation of stronger purifying selection in genes expressed broadly is in accordance with numerous previous studies (see, e.g., [27, 60]). The same is true for number of PPIs: Results of partial correlations between evolutionary rates and this property indicated that proteins with higher connectivity in PPI networks tend to evolve more slowly (681 genes: Spearman’s partial ρ = −0.177, *p* < 0.001; 1557 genes: Spearman’s partial ρ = −0.229, *p* < 0.001) (Figs. 2 and 3), a pattern described for various taxa (see, e.g., [56, 61, 62]). This connection might be ascribed to the greater portion of functional sites within highly connected proteins [24]. Additionally, deleterious mutations within proteins expressed in a wide range of tissues or having many interactors should have more serious consequences than such mutations in other proteins [27, 63]. This circumstance could also restrain their sequence evolution and might moreover underlie the correlations of tissue specificity and number of PPIs with essentiality (681 proteins; correlation between essentiality and τ: Spearman’s ρ = −0.318, *p* < 0.001; correlation between essentiality and number of PPIs: Spearman’s ρ = 0.256, *p* < 0.001; Fig. 1 and Additional file 1: Figure S1).

The relationship between dN/dS and survival essentiality was exclusively explored in the restricted dataset of 681 sperm protein-coding genes and we found a significant negative partial correlation (Spearman’s partial ρ = −0.194, *p* < 0.001; Fig. 2). In 1977 Wilson et al. [22] predicted that genes indispensable for survival or reproduction should evolve more slowly than nonessential genes. However, this postulate remained disputed after initial studies yielded contradictory results (see, e.g., [64, 65]), although the slower evolution of essential proteins could be confirmed in many following investigations (see, e.g., [34, 66]).

Overall, murine sperm genes which are indispensable for survival, broadly expressed, and/or whose encoded proteins are highly connected in PPI networks tend to evolve more slowly than other genes. Zero-order correlations highlight the interrelatedness among these variables (Fig. 1 and Additional file 1: Figure S1). Numbers of PPIs and gene essentiality are among the strongest independent rate correlates and τ appears to be an important determinant of dN/dS, too (Figs. 2 and 3); but beyond that, numbers of PPIs, survival essentiality, and expression breadth could influence evolutionary rate together with further properties as a compound factor which Choi and Hannenhalli [67] called the “function (fitness)-centred” variable.

### Expression level

Several studies found gene expression level to be an important determinant of evolutionary rates, especially in yeast [35, 37, 68, 69] and bacteria [36], but also in multicellular organisms [60, 70]. However, the correlation between rates of sequence evolution and expression level is rather weak in mammals [71], especially if other variables are controlled for [34] (see also [39]). Instead, breadth of expression seems to be a more prominent factor affecting mammalian protein evolution [34, 72]. Results of current analyses on murine sperm proteins underscore the greater importance of expression breadth than expression level in determining rates of protein evolution in mammalian species: While negative zero-order correlations between mean expression level of protein-coding genes and dN/dS were significant (681 genes: Spearman’s ρ = −0.259, *p* < 0.001; 1557 genes: Spearman’s ρ = −0.246, *p* < 0.001) (Table 1), the equivalent partial correlations became weaker (in the dataset of 1557 proteins; Spearman’s partial ρ = −0.060; *p* < 0.05; Fig. 3) or even nonsignificant (in the dataset of 681 proteins; Spearman’s partial ρ = −0.045; *p* > 0.05; Fig. 2). In contrast, the tissue specificity index τ was significantly positively correlated with evolutionary rate even when confounding factors were controlled for (see above; Figs. 2 and 3).

Feyertag et al. [73] discovered that the anticorrelation between evolutionary rate and expression level cannot be found in secreted proteins of diverse taxa. To investigate if the marginal or nonsignificant correlations between dN/dS and expression level observed in our two datasets relied on an enrichment of secreted proteins, we excluded these proteins and reran the analyses (see Additional file 1: Supplementary Methods). Results of partial correlations without secreted proteins remained qualitatively unchanged (see Additional file 1: Table S4). We conclude that our results are independent of the amount of secreted proteins. Hence, expression breadth remains the more relevant correlate of evolutionary rates in the murine sperm proteome compared with expression level.

### Evolutionary gene properties: Numbers of paralogs and gene age

Gene duplication may have two opposing effects on evolutionary rates. A phase of fast evolution seems to follow immediately after a duplication event, presumably due to relaxed constraints and/or the action of positive selection [38]. Later, if both copies are preserved in the genome increasing constraints [32] or functional relevance [38] account for a slow-down of sequence evolution. In our data, the latter effect was evident in significant negative partial correlations between the number of paralogs and dN/dS in both datasets (Figs. 2 and 3). This apparently reflects that many of the paralogs studied herein are rather ancient so that signatures of initial rate acceleration have already been superimposed by subsequent sequence conservation. The varying strength of correlation in the two datasets (681 genes: Spearman’s partial ρ = −0.162; *p* < 0.001; 1557 genes: Spearman’s partial ρ = −0.091; *p* < 0.001) might be a consequence of different sample compositions and sizes. However, the results altogether reveal a negative relationship between dN/dS and numbers of paralogs.

In the current study of murine sperm proteins, gene age also correlated negatively with dN/dS and was even among the strongest rate indicators according to partial correlation analyses (681 genes: Spearman’s partial ρ = −0.193, *p* < 0.001; 1557 genes: Spearman’s partial ρ = −0.183, *p* < 0.001) (Figs. 2 and 3). This agrees with findings by Albà and Castresana [32] who reported that older genes of mouse and human evolve more slowly than newer ones and proposed two models to explain this inverse relationship between evolutionary rates and gene age. Their model of “increasing constraint” predicts that beginning with weak selective pressures shortly after duplication, numbers of functional sites accumulate with time, thereby restraining evolutionary rates. According to their alternate model of “constant rate”, older evolutionary innovations (e.g., signal transduction or multicellularity) inherently entail more functional sites than newer ones. Neutral evolution should be more prevalent in younger genes containing fewer functionally constrained sites, resulting in higher evolutionary rates compared with older genes. Additionally, rapidly evolving genes are more likely to be lost from the genome and, thus, not to become “old”; therefore, they should be more numerous in younger age classes [32].

These theoretical considerations demonstrate that the possible mechanisms underlying the connections between rate of sequence evolution and both number of paralogs and gene age may be partly redundant. This overlap reflects the circumstance that probably most novel genes emerge from duplications [32, 74].

### Enrichment analyses

So far, we found that dN/dS estimates of murine sperm proteins correlate significantly with essentiality, protein connectivity, gene age, tissue specificity, number of paralogs, and UTR lengths, while we obtained contradicting or negative results for other variables (mean expression level, CDS length, average intron length, and multifunctionality). Another important factor influencing a protein’s sequence evolution whose exact impact is inaccessible through correlation analyses is its exact function (see, e.g., [13]). To gain insight into differences regarding the functions carried out by sperm proteins evolving at differing rates, we tested for enrichment of GO biological processes in three equally sized (each n = 519) protein groups classified according to their dN/dS estimates. Although we are aware that this procedure can only approximately assess influences of individual biological processes on evolutionary rate, it offered valuable clues. We used functional annotation clustering implemented in DAVID [50] to define clusters of related enriched terms within each of the three protein groups. Herein we present the top ten clusters for each protein class (Table 2), but all significantly enriched clusters (enrichment score ≥ 1.3; [50]) are specified in Additional file 2: Table S5. One result of these analyses was that the most rapidly evolving protein class was significantly enriched (enrichment score ≥ 1.3) with terms related to male reproduction and sperm-egg interaction, while the two more slowly evolving protein bins were not (Table 2). The enrichment of sperm-egg recognition terms accords with previous observations of most rapid evolution in sperm proteins operating within the female reproductive tract (see, e.g., [17, 18, 75]). Contrary to the two remaining protein classes, the group with highest dN/dS was additionally enriched for terms related to lipid metabolism and transport (Table 2). This functional enrichment could be associated with the alteration of the sperm plasma membrane’s lipid composition during epididymal transit, which might entail higher membrane fluidity [76]. Some enrichment clusters were similar in all three protein classes, such as those related to protein localization or sugar catabolism and we of course detected consistencies with the functional annotations reported in Chauvin et al. [40], including those corresponding to tricarboxylic acid (TCA) cycle and sugar metabolism (Table 2). In contrast to the most rapidly evolving protein groups, the two other protein classes were enriched with basal cellular processes such as protein complex assembly.

**Table 2:** Top 10 GO annotation clusters for three protein groups with different dN/dS estimates

“Low dN/dS”	“Medium dN/dS”	“High dN/dS”
Cluster description (enrichment score)	Cluster description (enrichment score)	Cluster description (enrichment score)
Protein localization (18.69)	Protein localization (7.84)	(Male) reproduction (5.99)
Protein localization (13.68)	tRNA metabolism (7.75)	Membrane lipid catabolism (3.51)
Macromolecular complex assembly (8.14)	TCA cycle (4.85)	Sperm-egg interaction (3.42)
(m)RNA processing (8.07)	ATP synthesis (3.95)	Sugar catabolism (3.11)
Protein catabolic process (7.85)	Porphyrin biosynthesis (3.39)	Response to oxidative stress (2.64)
Protein complex assembly (5.58)	Protein complex assembly (3.12)	Protein localization (2.31)
Regulation of protein complex assembly (3.71)	Sugar catabolism (2.91)	Porphyrin biosynthesis (2.09)
ATP synthesis (3.63)	Protein localization (2.89)	Regulation of lipid transport (2.02)
Sugar catabolism (3.59)	Cytoskeleton organization (2.43)	Lipid metabolism/catabolism (1.90)
Sugar metabolism (3.29)	(m)RNA transport (2.37)	Chemical homeostasis (1.79)

For a description of the three protein groups, see Methods. Names of clusters were assigned by the authors based on results of enrichment analyses with DAVID (version 6.7)

Taken together, results of GO analyses point to strongest sperm-specific functional enrichment in the most rapidly evolving protein class. Thus, proteins which evolve at relatively high rates are functionally more specialized than others in the murine sperm proteome. They should furthermore evolve under rather relaxed constraints as suggested by results of partial rank correlations. This outcome underscores that relaxation of constraints could be a prerequisite for adaptive evolution, especially in a sex-specific manner (see, e.g. [77]; see also [78, 79]).

## Conclusions

Based on our findings, we propose a model of sperm protein evolution taking into account rate correlates and functional aspects.

We conclude that most murine sperm proteins’ primary function is not reproduction-specific; instead, most members of the sperm proteome apparently engage in processes required in sperm as well as other cell types. Accordingly, results of GO enrichment analyses illustrate that only few of the 1557 proteins considered are engaged in testis- and sperm-specific processes. Furthermore, most sperm proteins display high phyletic ages, with more than two thirds of genes (1078 of 1557) assigned to the two oldest age classes. This high proportion of pre-metazoan proteins corresponds to the findings of Freilich et al. [80], whereupon a relatively constant fraction (~ 65%) of proteins in each of the 14 mouse tissues investigated in their study originated before the emergence of Metazoa. In the current study, genes which have been retained in the genome over long evolutionary timescales are more likely to be broadly expressed, have more PPI partners and paralogs, and to be essential for survival as depicted in Fig. 1 (see also Additional file 1: Figure S1). Results of our partial rank correlations highlight the respective properties – gene age, numbers of PPIs and paralogs, τ, and essentiality – as some of the strongest correlates of dN/dS of murine sperm proteins. This observation is largely consistent in both datasets (n = 1557 and n = 681; exception: essentiality which was only included in analyses of the smaller dataset). These results agree with the notion that slowly evolving genes are of “high status”, while rapidly evolving genes have “low status” [81], a pattern which, according to our analyses, apparently also applies to evolutionary rate distributions in the murine sperm proteome. Thus, most slowly evolving sperm proteins are constrained by their high status properties, such as a multitude of PPIs and paralogs, essentiality [81], and/or high age (see [74]), while proteins with lower status are potentially more susceptible to evolve neutrally or in response to natural, in some cases sexual, selection. Status differences might also be the basis of the functional and evolutionary compartmentalization detected in male reproductive proteins in various taxa, with proteins expressed at early stages of reproduction (spermatogenesis, sperm assembly) being more constrained than those involved in interaction with the female (sperm motility and sperm-egg interaction) ([17, 18]; see also [14]). The current study reinforces the notion of Dorus et al. [13] that conservation of sperm proteins might rely on their basal functions and/or their degree of pleiotropy. Moreover, we expand this view by factors such as phyletic gene age and interdependencies between the mentioned variables. Additionally, our results underline the importance of incorporating various gene properties when analysing the evolution of gene groups or genomes to gain a more general picture of factors underlying evolutionary rate variation. Furthermore, they emphasize that although some gene properties are general correlates of evolutionary rates, their importance may vary not only in a lineage-, but to some extent also in a cell type-specific manner.

## Additional Files

Additional File 1: **Figure S1**, **Supplementary Methods**, **Tables S1-S4**. Additional methods and results of additional analyses as specified in the main text. (PDF 429 kb)

Additional File 2: **Table S5**. Significantly enriched DAVID GO annotation clusters for three protein groups with different dN/dS estimates. (XLSX 17 kb)

## Abbreviations

CDS: coding sequence
DAVID: Database for Annotation, Visualization and Integrated Discovery
dN: nonsynonymous substitution rate
dS: synonymous substitution rate
GO: Gene Ontology
I2D: Interologous Interaction Database
ID: identifier
MGI: Mouse Genome Informatics
miRNA: microRNA
MP: Mammalian Phenotype
PPI: protein-protein interaction
TCA: tricarboxylic acid
UTR: untranslated region
τ: tissue specificity index
