# Supplementary material for: Correlates of evolutionary rates in the murine sperm proteome
Source: BMC Evol Biol. 2018 Mar 27;18:35. doi: 10.1186/s12862-018-1157-6 (PMC5870804; doi:10.1186/s12862-018-1157-6)
Supplement: Supplementary file 1 — Figure S1, Supplementary Methods, Tables S1-S4. Additional methods and results of additional analyses as specified in the main text. (PDF 429 kb) [file 12862_2018_1157_MOESM1_ESM.pdf]

Additional File 1 to "Correlates of Evolutionary Rates in the Murine Sperm Proteome"

by Julia Schumacher and Holger Herlyn

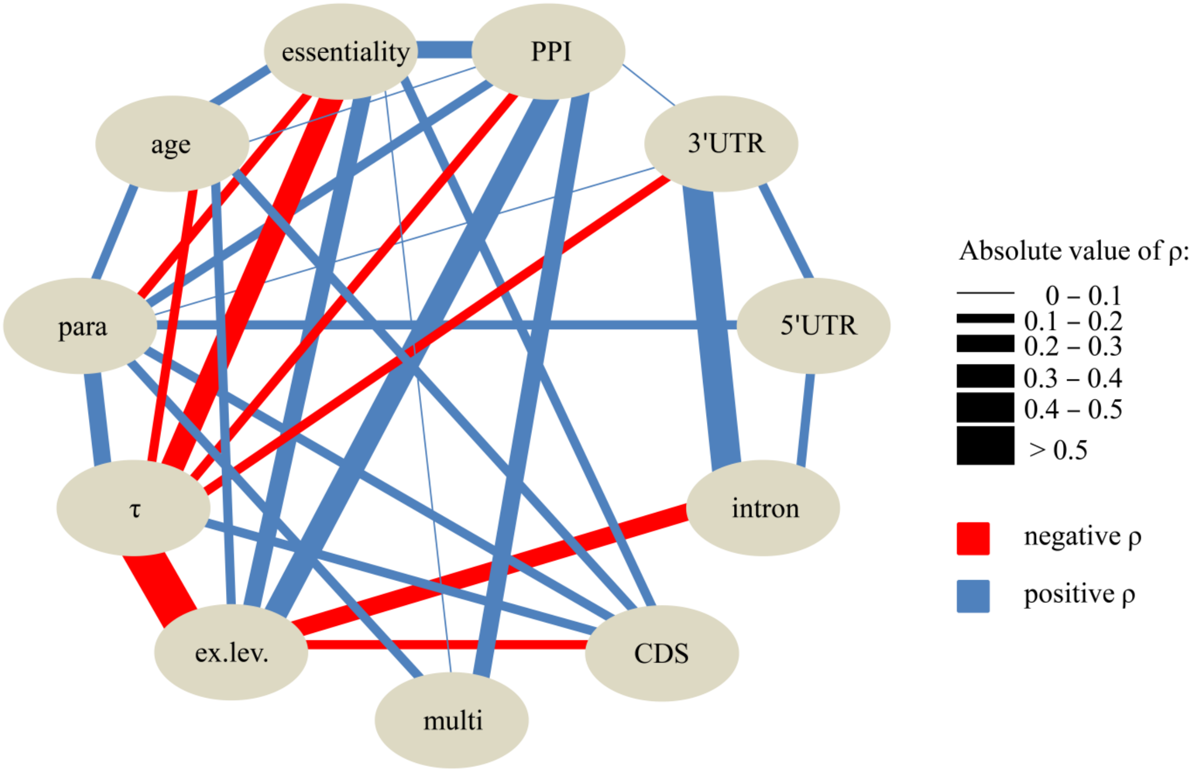

Fig. S1 Spearman's rank correlation between all variable pairs, excluding dN/dS. Edges represent correlation coefficients (ρ) between two properties. All correlations were calculated in the dataset of 681 proteins. Only correlations significant with $p < 0.05$ are depicted. age, phyletic age of genes; multi, multifunctionality (number of biological processes per protein according to GOSlim generic); PPI, number of PPIs; para, number of paralogs; CDS, coding sequence length; intron, average intron length; 3'UTR, length of 3' UTR; 5'UTR, length of 5' UTR; essentiality, essentiality for survival, 0 = nonessential, 1 = essential; τ, tissue specificity index; ex.lev., mean mRNA expression level.

Supplementary Methods

Mapping of sperm proteins to Ensembl Gene IDs

Swiss-Prot entry names extracted from supplementary table S1 of Chauvin et al. [1] were matched to their Swiss-Prot accession numbers using Uniprot's ID mapping tool (19th march 2015). We mapped the obtained Swiss-Prot accessions to Ensembl Gene IDs via Ensembl Biomart (version 79). If this procedure failed, we identified the proteins' Ensembl Gene IDs using gene names specified in supplementary table S1 of Chauvin et al. [1] or given in Uniprot. All Swiss-Prot accession numbers whose Uniprot entries denoted that more than one gene coded for the correspondent protein were excluded from the dataset (e.g. Swiss-Prot accessions P84228 or P62204). The same was true if Uniprot entries had been demerged into multiple entries. Moreover, several Swiss-Prot accession numbers were assigned to multiple Ensembl Gene IDs although only one gene was given in Uniprot. In such cases, we exclusively kept Ensembl Gene IDs on the murine primary genome assembly and with gene names corresponding to those reported in Uniprot, as we aimed to include only one Ensembl Gene ID per protein. The majority of the thereby removed Ensembl Gene IDs were referred to as pseudogenes, retrogenes, "predicted genes" (starting with "Gm") or "-related sequences" in their Ensembl entries. If several Ensembl Gene IDs identified for a single Swiss-Prot accession number had names corresponding to a gene symbol given in the Uniprot entry, but only one of them had an available dN/dS estimate for a 1-to-1 orthologue with rat (*Rattus norvegicus*; see main text), this Ensembl Gene ID was considered; if, however, more than one Ensembl Gene ID corresponding to a Swiss-Prot accession number had a dN/dS value (1-to-1 with rat), the protein was excluded. We furthermore ignored all proteins with Ensembl gene names which accorded to none of the gene symbols shown in their Uniprot entries. Finally, only genes designated as "protein_coding" according to Ensembl version 79 were retained in the dataset. Phospholipid hydroperoxide glutathione peroxidase (*Gpx4*) and Guanine nucleotide-binding protein G(s) subunit alpha (*Gnas*) were each represented by two different

Swiss-Prot entry names in the original data by Chauvin et al. [1], but were each kept as one Ensembl Gene ID in our dataset. However, to quantify numbers of PPI partners and multifunctionality (see main text and below) of these proteins we used the Swiss-Prot accession numbers P63094 (*Gnas*) and O70325 (*Gpx4*), for which entries were available in the PPI data used (see main text and below).

Number of paralogs, CDS length, average intron length, 5' and 3' UTR length

Numbers of paralogs per gene were derived from Ensembl Biomart (version 79) downloads. Canonical transcripts corresponding to each Ensembl Gene ID were retrieved via in-house Python scripts employing Pycogent library version 1.5.3 [2]. For each canonical transcript the coding sequence (CDS) as well as the sequences of 5' and 3' UTRs were extracted via Ensembl Biomart and their lengths were determined using Python scripts. Proteins for which at least one UTR of their canonical transcript was unavailable were excluded from further analyses. Average intron lengths of canonical transcripts were calculated with Python scripts utilizing Pycogent [2].

PPI data

When counting numbers of interactors in I2D data, we included only PPIs verified in mice and originally derived from BIND [3], BioGrid [4], DIP [5], InnateDB [6], IntAct [7], and MINT [8]. Self-interactions were discarded.

Biological process data

To count numbers of biological processes per protein, annotations from the GOA gene association file for mouse (state: 30th march 2015; downloaded from http://www.ebi.ac.uk/GOA/downloads) were mapped to GOSlim generic terms with the aid of map2slim. The nonredundant list of all biological process terms according to GOSlim was

included in the count, except for “GO:0008150” (biological_process) if given with the evidence code “ND”, which indicates absence of information (https://www.ebi.ac.uk/QuickGO/GTerm?id=GO:0008150).

Further partial rank correlation analyses

We recomputed partial Spearman’s rank correlations between the chosen gene properties and dN or dS. Additionally, partial correlation analyses as described in the main text were repeated with datasets after exclusion of genes with signals of positive selection and after exclusion of genes coding for secreted murine proteins.

Pairwise dN and dS values were extracted from Ensembl (version 79) Biomart and had been calculated for orthologous sequences of mouse and rat (see Materials and Methods of the main text) with CodeML from the PAML package [9].

Analyses of sequence evolution were conducted on alignments comprising sequences of mouse (*Mus musculus*, GRCm38.p3), rat (*Rattus norvegicus*, Rnor_5.0), human (*Homo sapiens*, GRCh38.p2), either chimpanzee (*Pan troglodytes*, CHIMP2.1.4) or macaque (*Macaca mulatta*, MMUL 1.0) (“primate”), and either dog (*Canis lupus familiaris*, CanFam3.1) or cow (*Bos taurus*, UMD3.1) (“Laurasiatheria”) (species sample as in [10]). For each species other than mouse, we identified Gene IDs for orthologous (1-to-1) genes of mouse using Ensembl (version 79) Biomart and extracted IDs of canonical transcripts using Pycogent [2]. We generated codon-based alignments with Clustal W 2.1 [11, 12] utilizing Pycogent [2] and BioPython [13]. Genes for which the species set could not be obtained were excluded from subsequent analyses. Alignments for *Gpx1* and *Gpx4* contained codons coding for selenocysteine in human and murine sequences. Since these codons are identified as stop in CodeML (see below), we deleted the respective positions from these two alignments. We tested for the presence of positively selected codon sites using CodeML implemented in PAML version 4.7 [14] based on the alignments and the following tree topology:

((mouse,rat),(human,primate),Laurasiatheria). For each gene, we carried out a likelihood ratio test (LRT) comparing the selection model M8 with the null model M8a [15].

When running the two models, ambiguity data were ignored (cleandata = 1). To avoid local optima, M8 analyses were run thrice with different initial ω values (0.6, 1.2, 1.6) and the least negative lnL of these three runs was used for LRT. Genes which showed significant ($p \leq 0.05$) signals of positive selection were excluded from the datasets (with or without the essentiality variable) before computing partial correlations.

Secreted murine proteins were identified based on data downloaded from MetazSecKB [16] using their Swiss-Prot accessions (see Materials and Methods section of the main text). As described in Feyertag et al. [17] we considered proteins as secreted if they were classified as either "Secreted (curated)" or "Secreted (highly likely)" in MetazSecKB. These secreted proteins were excluded from the datasets (with or without the essentiality variable) and partial correlations as described in the main text were recomputed.

References

1. Chauvin T, Xie F, Liu T, Nicora CD, Yang F, Camp DG2, et al. A systematic analysis of a deep mouse epididymal sperm proteome. Biol Reprod. 2012;87:141.
2. Knight R, Maxwell P, Birmingham A, Carnes J, Caporaso JG, Easton BC, et al. PyCogent: a toolkit for making sense from sequence. Genome Biol. 2007;8:R171.
3. Bader GD, Hogue CW. BIND--a data specification for storing and describing biomolecular interactions, molecular complexes and pathways. Bioinformatics. 2000;16:465–77.
4. Stark C, Breitkreutz B, Reguly T, Boucher L, Breitkreutz A, Tyers M. BioGRID: a general repository for interaction datasets. Nucleic Acids Res. 2006;34:D535-9.

5. Xenarios I, Rice DW, Salwinski L, Baron MK, Marcotte EM, Eisenberg D. DIP: The Database of Interacting Proteins. Nucleic Acids Res. 2000;28:289–91.
6. Lynn DJ, Winsor GL, Chan C, Richard N, Laird MR, Barsky A, et al. InnateDB: facilitating systems-level analyses of the mammalian innate immune response. Mol Syst Biol. 2008;4:218.
7. Aranda B, Achuthan P, Alam-Faruque Y, Armean I, Bridge A, Derow C, et al. The IntAct molecular interaction database in 2010. Nucleic Acids Res. 2010;38:D525-31.
8. Zanzoni A, Montecchi-Palazzi L, Quondam M, Ausiello G, Helmer-Citterich M, Cesareni G. MINT: a Molecular INTeraction database. FEBS Lett. 2002;513:135–40.
9. Yang Z. PAML: a program package for phylogenetic analysis by maximum likelihood. Comput Appl Biosci. 1997;13:555–6.
10. Dorus S, Wasbrough ER, Busby J, Wilkin EC, Karr TL. Sperm proteomics reveals intensified selection on mouse sperm membrane and acrosome genes. Mol Biol Evol. 2010;27:1235–46.
11. Thompson JD, Higgins DG, Gibson TJ. CLUSTAL W: improving the sensitivity of progressive multiple sequence alignment through sequence weighting, position-specific gap penalties and weight matrix choice. Nucleic Acids Res. 1994;22:4673–80.
12. Larkin MA, Blackshields G, Brown NP, Chenna R, McGettigan PA, McWilliam H, et al. Clustal W and Clustal X version 2.0. Bioinformatics. 2007;23:2947–8.
13. Cock PJA, Antao T, Chang JT, Chapman BA, Cox CJ, Dalke A, et al. Biopython: freely available Python tools for computational molecular biology and bioinformatics. Bioinformatics. 2009;25:1422–3.
14. Yang Z. PAML 4: phylogenetic analysis by maximum likelihood. Mol Biol Evol. 2007;24:1586–91.
15. Swanson WJ, Nielsen R, Yang Q. Pervasive adaptive evolution in mammalian fertilization proteins. Mol Biol Evol. 2003;20:18–20.

16. Meinken J, Walker G, Cooper CR, Min XJ. MetazSecKB: the human and animal secretome and subcellular proteome knowledgebase. Database (Oxford) 2015.

17. Feyertag F, Berninsone PM, Alvarez-Ponce D. Secreted Proteins Defy the Expression Level-Evolutionary Rate Anticorrelation. Mol Biol Evol. 2017;34:692–706.

Table S1 Spearman's rank correlations between dN and each gene property.

Gene properties	partial ρ with dN (n = 681)	partial ρ with dN (n = 1,557)
5'UTR	-0.095*	-0.104***
3'UTR	-0.164***	-0.127***
CDS	0.067 (ns)	0.104***
intron	0.031 (ns)	0.023 (ns)
multi	0.016 (ns)	0.048 (ns)
τ	0.148***	0.161***
PPI	-0.185***	-0.231***
essentiality	-0.184***	NA
ex.lev.	-0.043 (ns)	-0.050*
para	-0.150***	-0.086***
age	-0.183***	-0.180***

age, phyletic age of genes; multi, multifunctionality (number of biological processes per protein according to GOSlim generic); PPI, number of PPIs; para, number of paralogs; CDS, coding sequence length; intron, average intron length; 3'UTR, length of 3' UTR; 5'UTR, length of 5' UTR; essentiality, essentiality for survival, 0 = nonessential, 1 = essential; τ, tissue specificity index; ex.lev., mean mRNA expression level; NA, not applicable. Significance: * $p < 0.05$; *** $p < 0.001$; ns, nonsignificant.

Table S2 Spearman's rank correlations between dS and each gene property.

Gene properties	partial ρ with dS (n = 681)	partial ρ with dS (n = 1,557)
5'UTR	0.001 (ns)	-0.054*
3'UTR	-0.096*	-0.076**
CDS	0.068 (ns)	0.098***
intron	0.035 (ns)	-0.010 (ns)
multi	-0.057 (ns)	-0.001 (ns)
τ	0.152***	0.121***
PPI	-0.063 (ns)	-0.061*
essentiality	-0.022 (ns)	NA
ex.lev.	0.057 (ns)	0.029 (ns)
para	-0.009 (ns)	-0.000 (ns)
age	-0.008 (ns)	-0.030 (ns)

See legend of table S1 for 3'UTR, 5'UTR, age, essentiality, ex.lev., multi, para, PPI, CDS, intron, and τ. Significance: * $p < 0.05$; ** $p < 0.01$; *** $p < 0.001$; ns, nonsignificant.

Table S3 Spearman's rank correlations between dN/dS and each gene property after exclusion of positively selected genes.

Gene properties	partial ρ with dN/dS (n = 556)	partial ρ with dN/dS (n = 1,274)
5'UTR	-0.097*	-0.104***
3'UTR	-0.127**	-0.103***
CDS	0.067 (ns)	0.079**
intron	0.040 (ns)	0.036 (ns)
multi	0.028 (ns)	0.036 (ns)
τ	0.126**	0.124***
PPI	-0.178***	-0.232***
essentiality	-0.202***	NA
ex.lev.	-0.066 (ns)	-0.057*
para	-0.204***	-0.118***
age	-0.164***	-0.159***

See legend of table S1 for 3'UTR, 5'UTR, age, essentiality, ex.lev., multi, para, PPI, CDS, intron, and τ. Significance: * $p < 0.05$; ** $p < 0.01$; *** $p < 0.001$; ns, nonsignificant.

Table S4 Spearman's rank correlations between dN/dS and each gene property after exclusion of secreted proteins.

**Gene properties**	**partial ρ with dN/dS (n = 619)**	**partial ρ with dN/dS (n = 1,471)**
5'UTR	-0.095*	-0.104***
3'UTR	-0.132**	-0.098***
CDS	0.071 (ns)	0.098***
intron	0.054 (ns)	0.040 (ns)
multi	-0.003 (ns)	0.023 (ns)
τ	0.103*	0.128***
PPI	-0.190***	-0.237***
essentiality	-0.188***	NA
ex.lev.	-0.060 (ns)	-0.060*
para	-0.167***	-0.104***
age	-0.141***	-0.148***

See legend of table S1 for 3'UTR, 5'UTR, age, essentiality, ex.lev., multi, para, PPI, CDS, intron, and τ. Significance: * $p < 0.05$; ** $p < 0.01$; *** $p < 0.001$; ns, nonsignificant.
